# Risk factors associated with outcomes of peritoneal dialysis in Taiwan: An analysis using a competing risk model

**DOI:** 10.1097/MD.0000000000014385

**Published:** 2019-02-08

**Authors:** Hsiao-Ling Chen, Der-Cherng Tarng, Lian-Hua Huang

**Affiliations:** a Department of Nursing, Taipei Veterans General Hospital, School of Nursing, College of Medicine, National Taiwan University; b Division of Nephrology, Department of Medicine, Taipei Veterans General Hospital, Department and Institute of Physiology, National Yang-Ming University; c Professor, School of Nursing, China Medical University, Emeritus Professor, School of Nursing, National Taiwan University, Taiwan.

**Keywords:** Diabetes mellitus, gender difference, peritoneal dialysis, peritonitis, technique failure

## Abstract

Peritoneal dialysis (PD) is one option for renal replacement therapy in patients with end-stage renal disease (ESRD). Maintenance of the PD catheter is an important issue for patient outcomes and quality of life. The aim of this retrospective cohort study is to clarify the risk factors of technique failure and outcomes at a single institute in Taiwan.

The study enrolled ESRD patients who had received PD catheters in a tertiary hospital in northern Taiwan. Using a competing risks regression model, we reviewed clinical data and analyzed them in terms of the time to technical failure and clinical outcomes, including PD-related peritonitis and mortality.

A total of 514 patients receiving PD between 2001 and 2013 were enrolled in the study. According to the multivariate analysis model, we found that diabetes mellitus was a risk factor for PD-related peritonitis (subdistribution hazard ratio [SHR] 1.47, 95% confidence interval [CI] 1.06–2.04, *P* = .021). Female gender and higher serum albumin levels were associated with lower risks of technique failure (SHR 0.67, 95% CI 0.48–0.94, *P* = .02; SHR 0.75, 95% CI 0.58–0.96, *P* = .023, respectively), but Gram-negative and polymicrobial infection increased the technique failure rate (SHR 1.68, 95% CI 1.08–2.61, P = .021; SHR 1.93, 95% CI 1.11–3.36, P = .02, respectively). Female gender was a risk factor associated with overall mortality (SHR 6.4, 95% CI 1.42–28.81, *P* = .016). Higher weekly urea clearance (Kt/V) and weekly creatinine clearance (WCCr) were associated with a lower risk of mortality (SHR 0.1, 95% CI 0.01–0.89, *P* = .04; SHR 0.97, 95% CI 0.96–0.99, *P* = .004, respectively).

Diabetes mellitus is a risk factor contributing to PD-related peritonitis. Male patients and lower serum albumin levels were associated with higher rates of technique failure. Female gender, lower Kt/V, and WCCr are risk factors for overall mortality in PD patients.

## Abstract 2

Supplemental Digital Content is available in the text

## 1 Introduction

Taiwan has the highest incidence and greatest prevalence of end-stage renal disease (ESRD) according to an international comparison based on an annual report from the United States Renal Data System.^[1]^ In 2015, the incidence of ESRD was 476 per million people in Taiwan, and the prevalence of treated ESRD was 3317 per million people. End-stage renal disease has major impacts on public health by increasing medical expenses and financial burdens. End-stage renal disease patients represent 0.37% of the total population, but the annual expenditure for their treatment accounts for 6% of the total National Health Insurance (NHI) budget in Taiwan.^[2]^

Both peritoneal dialysis (PD) and hemodialysis (HD) are options for renal replacement therapy for patients with ESRD, aside from kidney transplantation. However, the majority of patients worldwide receive HD, which accounts for 89% of cases.^[3]^ Peritoneal dialysis and HD have similar survival results despite of PD being associated with lower medical costs in developed countries. In Taiwan, HD is more popular than PD, and only around 10% of ESRD patients receive PD.^[4]^ Nevertheless, the total lifetime cost for PD patients is lower than that for HD patients (estimated at about US $139,360 vs $185,235, respectively).^[5]^

The reasons for choosing HD rather than PD include a fear of developing peritonitis, which has been one of the major causes of death among PD patients in Taiwan, as well as worries about self-care procedures.^[5]^ The occurrence of complications such as PD-related peritonitis may result in further withdrawal from PD and even mortality.^[6]^ There is limited understanding in regard to who is most at risk for loss of PD catheter function and technique failure in Taiwan. To identify the risk factors associated with PD outcomes, further improvement in medical care and reducing complications are essential.

Both renal transplantation and switching to HD may hamper the observation of events when analyzing the survival data of PD patients.^[7]^ A competing risk is an event that hinders the observation of an event of interest or modifies its probability of occurrence. In these situations, it is not adequate to apply standard survival models and methods like Cox regression to the cause-specific hazard. Rather, the analysis should involve a competing risk model to identify the risk or survival of PD patients.^[8,9]^ The objective of this study is to clarify the risk factors associated with the technique failure of PD catheters and related outcomes, including PD-related peritonitis and mortality. This retrospective observational cohort study was conducted at a single institute in Taiwan.

## 2 Methods

### 2.1 Eligible patients

The study population consisted of patients who received a PD catheter and dialysis at Taipei Veterans General Hospital (VGH). This hospital is a tertiary hospital in northern Taiwan and a major organ transplantation center with a multidisciplinary team of doctors, nurses, dieticians, case managers, coordinators, and other clinicians. The study protocol was approved by the Institutional Review Board of VGH (Clinical Trial/Research Approval No 2016-02-007CC). The study proceeded from January 1, 2001 to December 31, 2013. Nephrologists chose willing candidates with PD for renal replacement therapy. The types of PD included both continuous ambulatory peritoneal dialysis (CAPD) and automated peritoneal dialysis (APD).

The PD insertion procedures all involved the conventional open access technique with either spinal or general anesthesia. In all cases, patients received prophylactic antibiotics with 1 g of intravenous cefazolin prior to the placement of a Tenckhoff catheter. The surgeon made a paramedian skin incision on the right side except when considering the adhesion of the right peritoneal cavity, such as when there was a history of appendectomy or ascending colonic diverticulitis.

The surgeon made a small opening in the fascia of the rectus sheath and peritoneum. The catheter tip was placed near the pelvic cavity using a stiff guide wire, and the position was confirmed using fluoroscopy. The catheter exited from the lower-right quadrant with a tunneled segment inside the rectus sheath and subcutaneous tissue. The patient initiated PD within a break-in period of 3 days to 1 week after the surgical placement of the catheter. The standard PD training program for patients lasted for 5 days.

### 2.2 Risk factors

We reviewed clinical data and medical records retrospectively. The data included age, gender, body mass index (BMI), medical comorbidities, and results of a laboratory examination, including nutritional parameters (serum albumin and lipid profiles). We recorded the causative pathogens in peritonitis according to the bacteriologic culture of the dialysate or ascites. We calculated the Charlson Comorbidity Index (CCI) for each patient.^[10]^ We considered patients to have cerebrovascular disease (CVD) if they had a history of stroke, cerebrovascular events, transient ischemic attack, intracranial aneurysms, or vascular malformations.^[11]^ We considered patients to have hyperlipidemia if they had hypercholesterolemia (which was noted if serum total cholesterol >200 mg/dL or low-density lipoprotein cholesterol (LDL) >130 mg/dL) or hypertriglyceridemia (>150 mg/dL).^[12,13]^ We estimated the residual renal function (RRF) by calculating the creatinine clearance and recorded data about peritoneal function, such as total weekly urea clearance (Kt/V), weekly clearance of creatinine (WCCr), and results of the peritoneal equilibration test (PET).

### 2.3 Clinical outcomes

We recorded the dates of outcome events for each patient, including the insertion of the PD catheter, first admission due to PD-related peritonitis, removal of the PD catheter, kidney transplantation, and death. The survival data comprised overall mortality with all causes. We obtained the times to outcome events, including withdrawal of PD the catheter, PD-related peritonitis, and death. We only analyzed data from the first catheter placements for patients who had more than one placement during the study period. We excluded patients who began PD after renal allograft failure. The basis of peritonitis diagnosis is the presentation of any two of the following symptoms: abdominal pain, turbid dialysate with a white blood cell count more than 100/mL and 50% neutrophils, or a positive peritoneal fluid culture.^[14]^ We also took into account the causative pathogens for the outcome analysis.

### 2.4 Statistical analysis

We present data using absolute frequencies and percentages for categorical variables and the mean ± standard deviation (SD) for continuous variables. We estimated survival data using the intention-to-treat approach. We used a competing risks regression model to analyze outcomes.^[8]^ We considered kidney transplantation or removal of the PD catheter before a death event as a competing risk for analyzing mortality outcomes. We considered patients who had a death event without peritonitis or received kidney transplantation without prior peritonitis as having a competing risk for the outcomes of peritonitis. We regarded renal transplantation or a death event without removing the PD catheter as a competing risk for the outcomes of withdrawing from PD.

We used a univariate analysis model to investigate the relationship between each independent variable. We used a multivariate analysis model to determine the independent variables that continued to have associations with outcomes after including significant variables in the univariate analysis. We calculated the subdistribution hazard ratios (SHR) of the covariates of competing risks regression with the respective 95% confidence intervals (CI). We analyzed the data using Stata 12.0 software (StataCorp LP, College Station, TX, USA), and we considered *P* values less than .05 as being statistically significant.

## 3 Results

### 3.1 Study population

**Figure 1:**
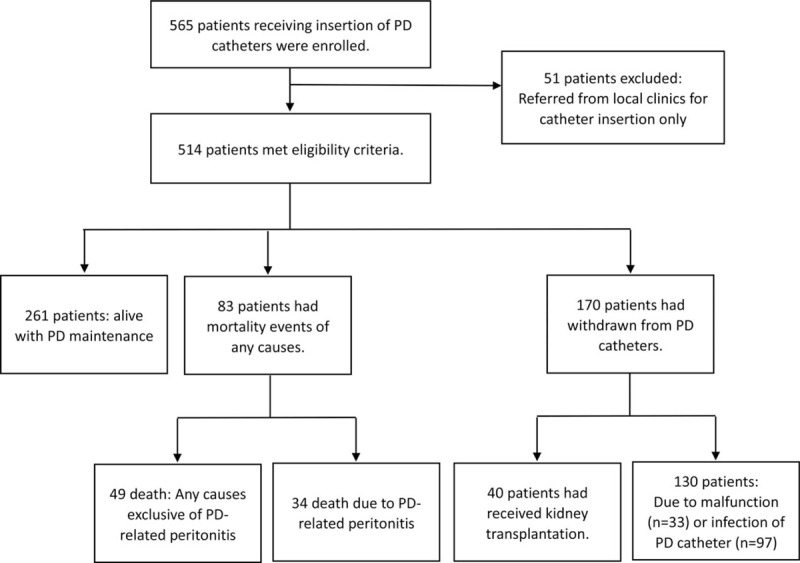
Flowchart of the study design and population.

The analysis initially included a total of 565 patients who received PD catheters between 2001 and 2013. We excluded 51 patients because they received referrals from local clinics and did not undergo follow-up at our hospital. Therefore, we included 514 patients in the final analysis (Fig. 1). The mean age of the cohort was 53.8 ± 16.0 years (range: 16–88 years). The median follow-up duration was 21.3 months (range: 0.3–132.2 months). Table 1 lists the demographic characteristics of the study patients. There were 77 patients who used APD (15%) as their dialysis modality; the remaining 437 patients used CAPD (85%).

**Table 1:**
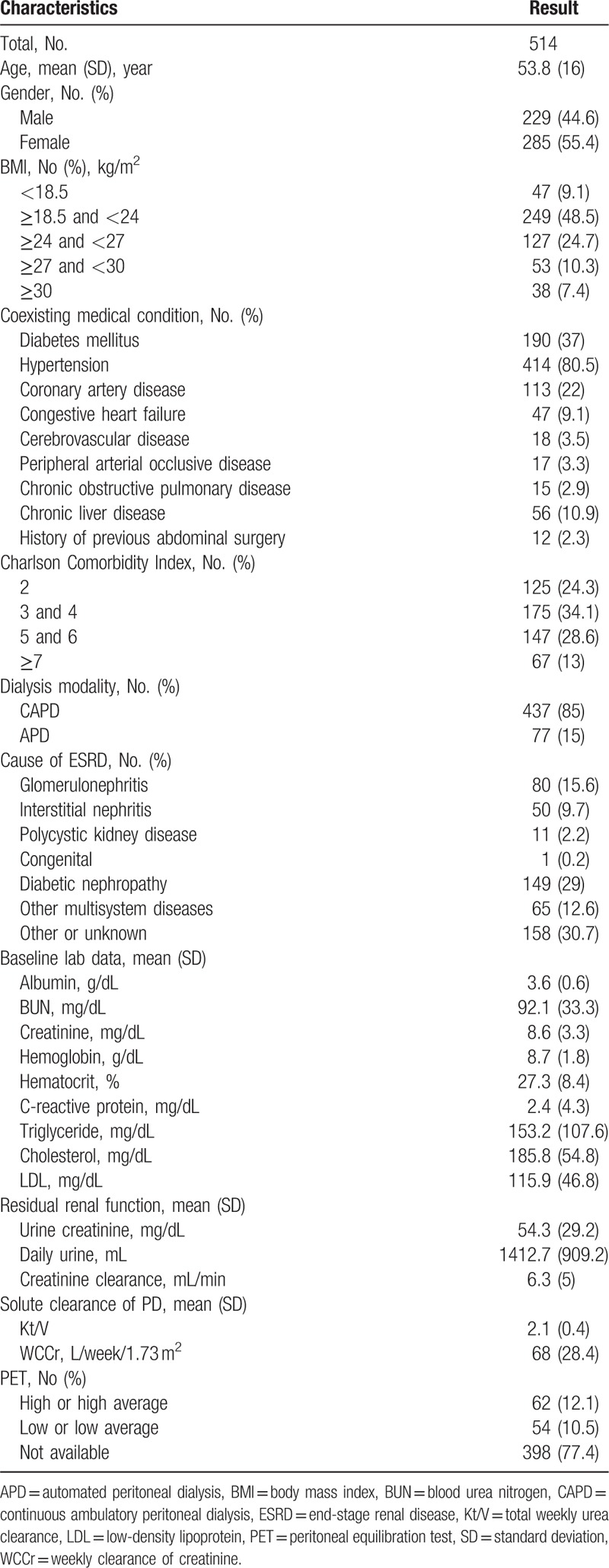
Demographic characteristics of patients.

### 3.2 Outcomes of PD-related peritonitis

More than half of the patients (n = 267, 50.8%) had episodes of PD-related infection, and 240 of them had PD-related peritonitis (46.7% of the total). The incidence of PD-related peritonitis was 0.18 cases per person-year. Diabetes mellitus (DM), congestive heart failure, higher CCI, and hypoalbuminemia had a significant association with PD-related peritonitis in the univariate competing risk model (SHR 1.74, 95% CI 1.35–2.24, *P* < .001; SHR 1.54, 95% CI 1.00–2.35, *P* = .048; SHR 1.12, 95% CI 1.05–1.19, *P* = .001; and SHR 0.77, 95% CI 0.63–0.94, *P* = .011, respectively) (Table 2).

**Table 2:**
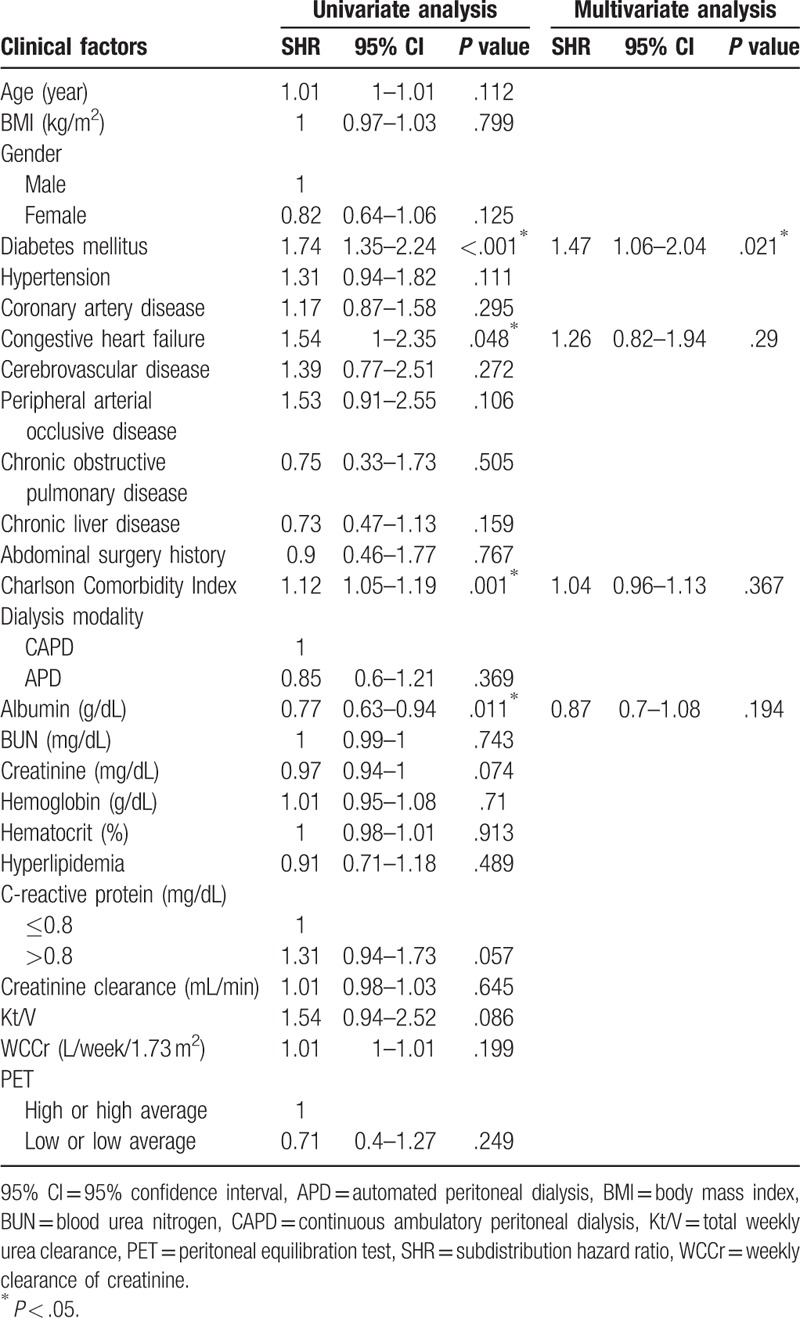
Risk factors associated with peritonitis related to peritoneal dialysis using competing risk analysis.

**Figure 2:**
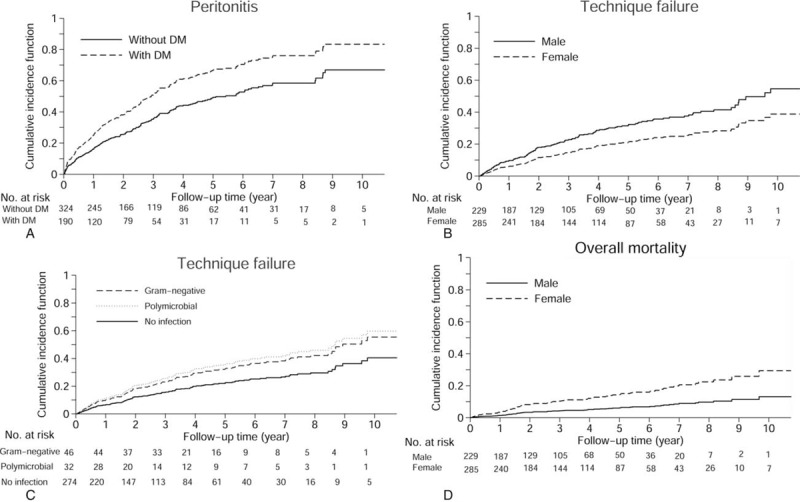
Cumulative incidence function of outcomes in patients with peritoneal dialysis: (A) peritonitis related to peritoneal dialysis between diabetic and non-diabetic patients, (B) technique failure between male and female patients, (C) technique failure among the Gram-negative, polymicrobial infection, and non-infection groups, and (D) overall survival of male and female patients.

In the multivariate analysis model, DM was the only statistically significant risk factor associated with PD-related peritonitis (SHR 1.47, 95% CI 1.06–2.04, *P* = .021). Figure 2A shows the cumulative incidence function (CIF) curves between diabetic and non-diabetic patients, and Table 3 shows the distributions of the causative pathogens of PD-related peritonitis. Gram-positive bacteria accounted for 32.6% of the bacteriologic cultures as the most common pathogen of infection, and Gram-negative bacteria accounted for 19.1% of the bacteriologic cultures. Polymicrobial infection accounted for 13.3% of the bacteriologic cultures. The distributions of bacteriologic culture between non-diabetic and diabetic patients are shown in the appendix.

**Table 3:**
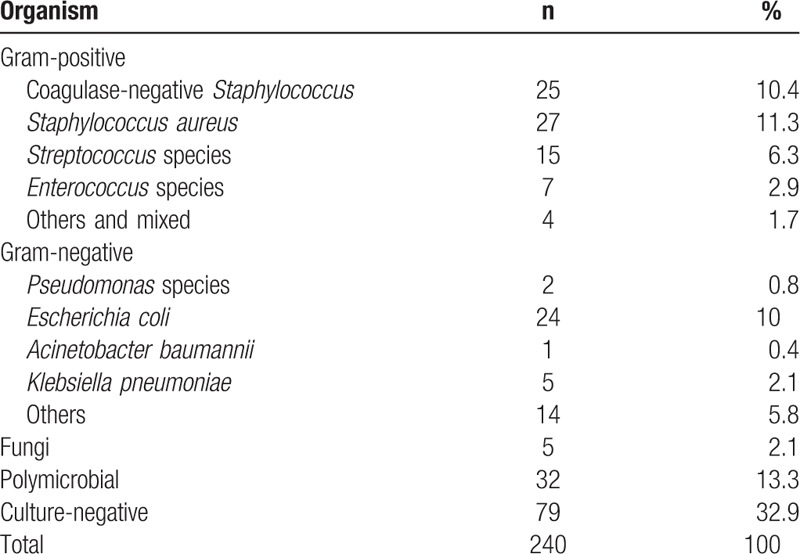
Causative pathogens in peritonitis related to peritoneal dialysis.

### 3.3 Outcomes of technique failure

One hundred and seventy patients withdrew from PD (33.1%). After excluding the 40 patients who received renal transplantation, technique failure occurred in 130 patients, and the physician removed their PD catheters due to either PD-related infection or malfunction of the catheters. Among the patients with technique failure, 97 experienced PD-related infections. The physician ceased PD in 78 patients due to peritonitis (80.4%); 13 patients had tunnel infection, and 6 patients had exit-site infection (13.4% and 6.2%, respectively).

In the univariate and multivariate competing risk model, female patients had a lower risk of technique failure than males (SHR 0.67, 95% CI 0.48–0.94, *P* = .02) (Table 4). Figure 2B shows the CIF curves of technique failure between male and female patients. Higher serum albumin levels were associated with a lower risk of technique failure in the univariate and multivariate models (SHR 0.75, 95% CI 0.58–0.96, *P* = .023). Gram-negative and polymicrobial infection were also associated with a higher rate of technique failure (SHR 1.68, 95% CI 1.08–2.61, *P* = .021; SHR 1.93, 95% CI 1.11–3.36, *P* = .02, respectively). Figure 2C shows the CIF curves of technique failure among the Gram-negative, polymicrobial infection, and non-infection groups.

**Table 4:**
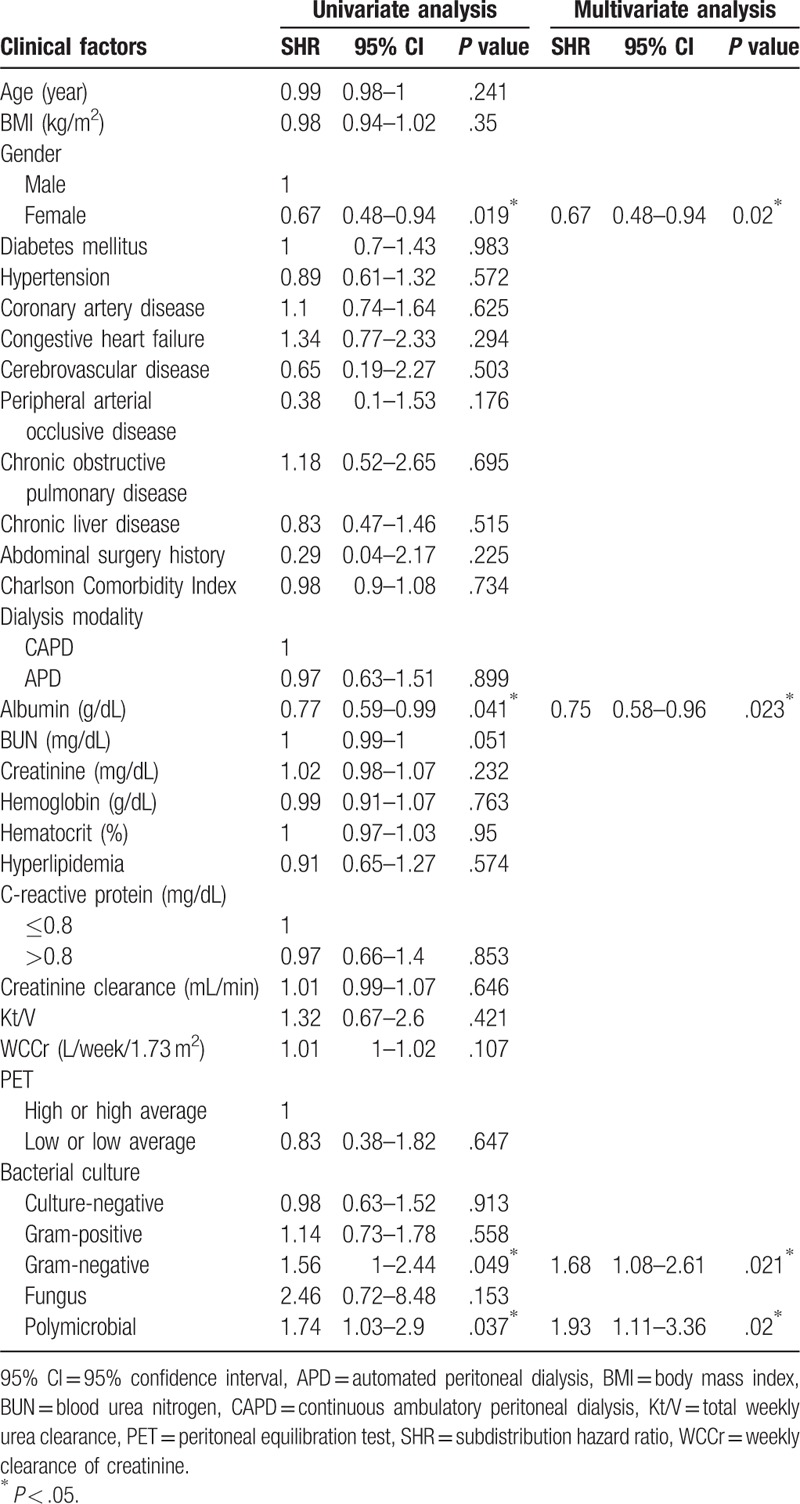
Risk factors associated with technique failure using competing risk analysis.

### 3.4 Outcomes of mortality

During the study period, 83 patients (16.1%) had mortality events, including all causes. Among these mortality cases, 34 resulted from peritonitis (41%). In the univariate analysis of the competing risk model, the risk factors associated with overall mortality were age, female gender, higher CCI, DM, coronary artery disease (CAD), congestive heart failure, CVD, lower serum albumin level, serum creatinine level, higher serum C-reactive protein level, lower total weekly urea and creatinine clearance (Kt/V and WCCr) (Table 5). In the multivariate analysis model, female gender was a risk factor associated with overall mortality (SHR 6.4, 95% CI 1.42–28.81, *P* = .016). Higher Kt/V and WCCr were associated with a lower risk of mortality (SHR 0.1, 95% CI 0.01–0.89, *P* = .04; SHR 0.97, 95% CI 0.96–0.99, *P* = .004, respectively). Figure 2D shows the CIF curves of overall mortality for gender.

**Table 5:**
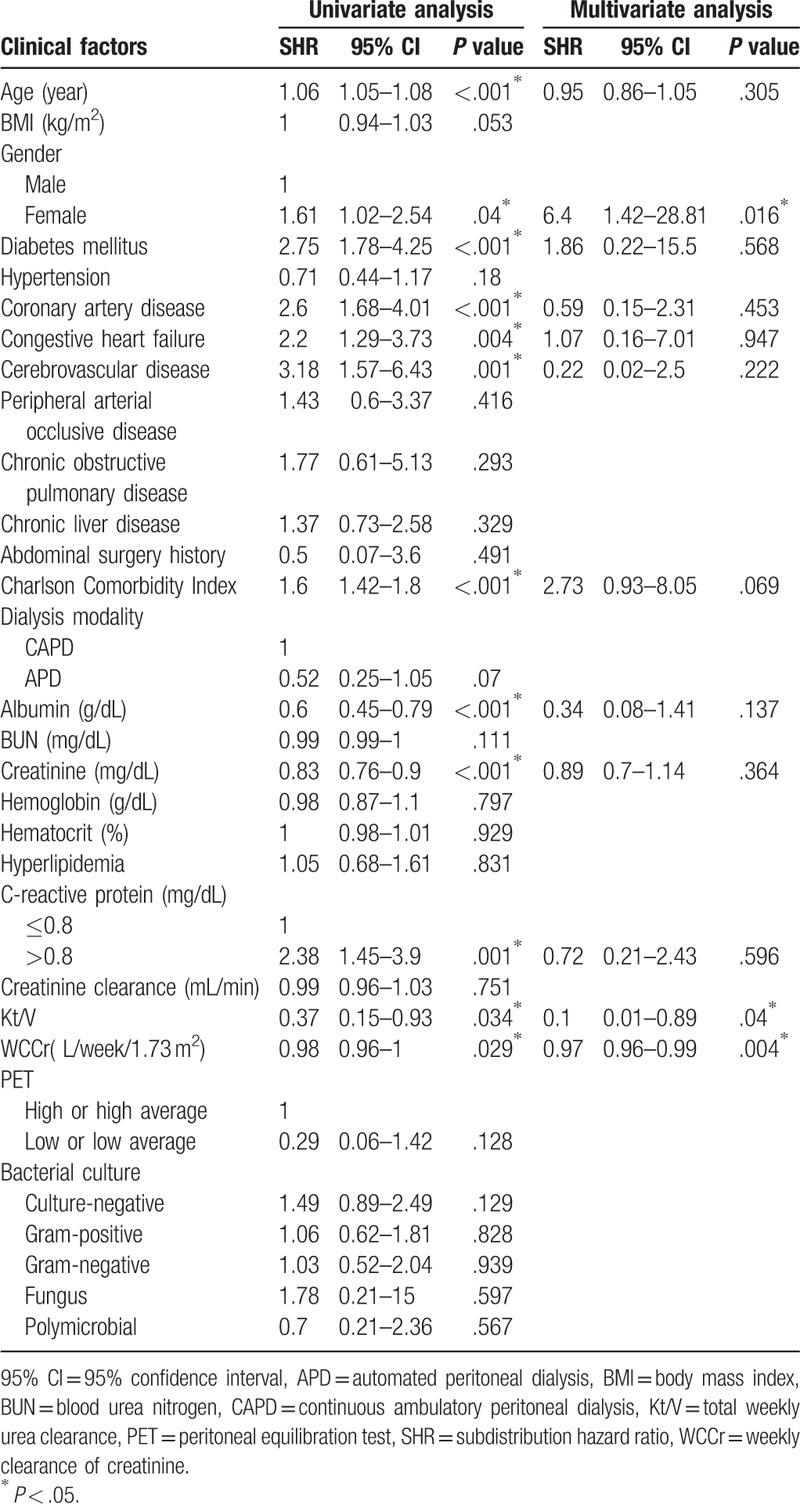
Risk factors associated with overall mortality using competing risk model.

## 4 Discussion

The number of patients with ESRD is increasing and this population constitutes a growing burden on public health and healthcare systems in Taiwan. The estimated life expectancy of patients with HD and PD is nearly equal, but the average lifetime healthcare costs are higher for HD than PD.^[5,15]^ In Taiwan, the NHI budget fully covers the total cost of dialysis and related treatment without deductibles or copayments for ESRD patients. In addition, the coverage rate of the NHI healthcare program is 99.9% of the population. Therefore, we do not consider personal economic hardship as an influencing factor in choosing the dialysis modality. However, only roughly 10% of patients with ESRD select PD as their renal replacement therapy.^[4]^ The possible influential factors in this decision include self-care ability and personal knowledge about dialysis modalities.^[16]^

Female gender was a risk factor for technique failure in a previous report^[17]^; another report indicated no gender differences in technique failure.^[18]^ In our study, however, male gender was a risk factor for technique failure than female gender. In our institute, the selection of renal replacement therapy with either HD or PD depends on the patient's willingness and the physician's evaluation. All of our patients with PD followed the same treatment protocol and training program. Selection bias may conceal the true mechanism behind this phenomenon, and it is not possible to perform a randomized trial, which would be ideal.

The possible mechanism of this gender difference may be due to biological or behavioral factors. For instance, there may be innate gender differences between male and female patients, such as anatomical structures of the peritoneal cavity and sex hormones. For example, males have peritoneum intake and a completely closed peritoneal cavity. In contrast, females usually have a potential pathway between the genital tract and the peritoneal cavity. However, our results show that male patients had a higher rate of technique failure than females, despite the advantage of biological factors.

The individual characteristics of self-care such as adherence or illness perception also warrant attention.^[19]^ Several disease outcomes have gender differences due to behavioral factors, such as periodontal status, oral hygiene,^[20,21]^ and diet control among diabetic patients.^[22–24]^ For instance, Schulze et al investigated gender differences between diabetic patients and non-diabetic patients by comparing periodontal status and oral hygiene.^[20]^ Their results indicate that men had worse periodontal status than women. Poor oral self-care correlated with poor periodontal status. Other studies have also revealed better oral hygiene care among adult women.^[25]^ Nevertheless, the relationships between self-care and gender require further investigation for PD patients.

Peritonitis is the primary complication of PD and remains the leading cause of hospitalization and technique failure.^[6]^ In our series, half of the patients experienced an episode of peritonitis, and 60% of the technique failures resulted from peritonitis. In addition, peritonitis was responsible for 41% of cases of all-cause mortality. There are various risk factors for peritonitis, such as DM,^[26]^ older age,^[27]^ indigenous race,^[28]^ and female sex.^[29]^ However, DM was the only risk factor for PD-related peritonitis in our patients. Although we do not preclude diabetic patients from selecting PD as a renal replacement modality, meticulous care is essential for these patients to avoid peritonitis and related complications.

Our study showed that Gram-positive bacteria were the most common causative organisms in 32.6% of peritonitis cases, and *Staphylococcus aureus* was the most common species causing the first peritonitis episode. However, Gram-negative bacteria and polymicrobial infection were associated with a higher risk of technique failure than other pathogens in our series. Although fungal peritonitis is rare among the causative organisms, it usually leads to serious consequences.^[30,31]^ Our results did not show statistical significance of technique failure for fungal peritonitis (SHR 2.46, 95% CI 0.72–8.48, *P* = .153, Table 4). This situation was most likely due to the small number of cases in our series (n = 5) or the incidence of fungal peritonitis being underestimated because of a negative culture. The distribution of causative pathogens varies between different series and countries. In an Australian series, Enterococcal peritonitis was associated with an increased risk of technique failure and death.^[32]^ In an Indian series, Gram-negative peritonitis was more frequent than Gram-positive peritonitis and was associated with increased technique failure.^[33]^ Adequate selection of empirical antibiotics is essential for the initial treatment of PD infection.^[14]^ It is necessary to perform clinical trials for primary and secondary prevention of PD infection.

In addition, our results showed that lower serum albumin levels were associated with an increased risk of technique failure. Albumin level is correlated with some liver disease, nephrotic syndrome, malabsorption, and malnutrition.^[34]^ Another study has shown that albumin is associated with an increased risk for mortality and technique failure.^[35]^ It is important to evaluate and improve the patients’ nutrition status for PD care.

Various studies have also reported risk factors that contribute to mortality in PD patients.^[36,37]^ Our study revealed that the risk factors for mortality among PD patients were female gender and poor solute clearance of peritoneal function (Kt/V and WCCr). Some studies have shown that residual renal function is associated with mortality,^[38]^ but our results revealed that poor solute clearance was associated with increased overall mortality. In a study conducted in the Netherlands, the authors demonstrated that peritoneal ultrafiltration was significantly associated with patient survival.^[39]^

Women usually have a longer life expectancy than men in the general population, but female gender was found to be a significant risk factor rather than a protective factor for mortality among PD patients in our series (SHR 6.4). The actual mechanism for the disparity between the lower rate of technique failure and higher mortality rate of female patients was not clear. Postmenopausal female patients may be more likely to develop cardiovascular disease compared with males.^[37]^ Women on dialysis usually experience menopause roughly 5 years earlier than the general population.^[40]^ Hypothalamic-pituitary-gonadal axis dysfunction may also impair sex hormones and the immune system. It may contribute to an increased risk of infection and malignancy in younger females undergoing dialysis.^[41]^ The median age of our female patients was 51 years, which we assumed to imply menopause. We hypothesized that the dysfunction of sex hormones in female patients with PD might increase cardiovascular risk (including prior or de novo CAD) and decrease the immune function of the host. Therefore, dysfunction of sex hormones resulted in an increased risk of overall mortality for female patients compared with males. There is still room for further investigation of this aspect. This finding should not drive changes in practice yet (i.e., prevent female patients from choosing PD as their dialysis modality).

The limitations of our study are its retrospective design and the fact that its population was drawn from a single institute. Most of the PET data were not recorded or tested in our series. The statistical power of detecting endpoint difference between various levels of PET may be low due to the relatively small cohort size. Nevertheless, our patients all received the same surgical insertion procedures and followed a standard care protocol for PD catheters. Furthermore, we adjusted for possible influential factors using a competing risk model. Additional multicenter studies might reveal more accurate risk factors for the general population.

In conclusion, a male gender is associated with more technique failures of PD catheters than a female gender. Diabetes mellitus was the only risk factor for PD-related peritonitis. Female gender was a risk factor for mortality among PD patients. Females have longer PD catheter usage, but this usage adversely affects survival. Gender differences may offer information for patients and healthcare practitioners making decisions related to the maintenance of PD catheters.

## Acknowledgments

The authors are grateful to Ms. Hsin-Yi Huang of the Biostatistics Task Force of Taipei Veterans General Hospital for assistance with the statistical analyses.

## Author contributions

**Conceptualization:** Hsiao-Ling Chen.

**Data curation:** Hsiao-Ling Chen.

**Formal analysis:** Der-Cherng Tarng, Lian-Hua Huang.

**Investigation:** Hsiao-Ling Chen.

**Methodology:** Der-Cherng Tarng.

**Software:** Hsiao-Ling Chen, Der-Cherng Tarng.

**Supervision:** Der-Cherng Tarng, Lian-Hua Huang.

**Validation:** Der-Cherng Tarng, Lian-Hua Huang.

**Writing – original draft:** Hsiao-Ling Chen.

**Writing – review & editing:** Der-Cherng Tarng, Lian-Hua Huang.

## Supplementary Material

Supplemental Digital Content
