# Supplementary material for: Risk factors associated with outcomes of peritoneal dialysis in Taiwan: An analysis using a competing risk model
Source: Medicine (Baltimore). 2019 Feb 8;98(6):e14385. doi: 10.1097/MD.0000000000014385 (PMC6380716; doi:10.1097/MD.0000000000014385)
Supplement: Supplemental Digital Content [file medi-98-e14385-s001.docx]

**Supplementary Table 1**

Demographic characteristics of the excluded patients.

| Characteristics | Number | % |
| --- | --- | --- |
| Total | 51 | 100 |
| Gender |  |  |
| Male | 28 | 54.9 |
| Female | 23 | 45.1 |
| BMI (kg/m^2^) |  |  |
| <18.5 | 2 | 3.9 |
| ≥18.5 and <24 | 41 | 80.4 |
| ≥24 and <27 | 8 | 15.7 |
| ≥27 and <30 | 0 | 0 |
| ≥30 | 0 | 0 |
| Diabetes mellitus |  |  |
| Yes | 9 | 17.6 |
| No | 42 | 82.3 |
| Hypertension |  |  |
| Yes | 19 | 37.3 |
| No | 32 | 62.7 |
| Coronary artery disease |  |  |
| Yes | 8 | 15.7 |
| No | 43 | 84.3 |
| Cerebrovascular disease |  |  |
| Yes | 2 | 3.9 |
| No | 49 | 96.1 |
| Respiratory disease |  |  |
| Yes | 0 | 0 |
| No | 51 | 100 |
| Liver disease |  |  |
| Yes | 2 | 3.9 |
| No | 49 | 96.1 |
| History of previous abdominal surgery |  |  |
| Yes | 0 | 0 |
| No | 51 | 100 |
| Charlson comorbidity index |  |  |
| 2 | 11 | 21.5 |
| 3 and 4 | 21 | 41.2 |
| 5 and 6 | 8 | 15.7 |
| ≥7 | 11 | 21.6 |

BMI = body mass index.

Note: The mean age was 53.9±16 years (range: 29 to 91 years). We did not list the ratios of automatic or continuous ambulatory peritoneal dialysis because of lost follow-up at our institute.

**Supplementary Table 2**

Distribution of bacteriologic culture from ascites or dialysate between nondiabetic and diabetic patients with peritonitis.

| Organism | Non-diabetic | | Diabetic | | Total | |
| --- | --- | --- | --- | --- | --- | --- |
|  | n | % | n | % | n | % |
| Gram-positive bacteria | 33 | 25 | 45 | 41.7 | 78 | 32.5 |
| Gram-negative bacteria | 30 | 22.7 | 16 | 14.8 | 46 | 19.2 |
| Fungi | 3 | 2.3 | 2 | 1.9 | 5 | 2.1 |
| Mixed culture | 15 | 11.4 | 17 | 15.7 | 32 | 13.3 |
| Culture-negative | 51 | 38.6 | 28 | 25.9 | 79 | 32.9 |
| Total | 132 | 100 | 108 | 100 | 240 | 100 |

Fisher’s exact test *P* = .024

**Supplementary Figure 1**

Cumulative incidence function of technique failure among different serum albumin level
